# Cross-Sectional Study on Electrocardiographic Disorders in Patients with Ankylosing Spondylitis in Real-World Conditions

**DOI:** 10.3390/jcm15010362

**Published:** 2026-01-03

**Authors:** Carlos Rodríguez-López, Bárbara Soler Bonafont, Álvaro Gamarra, Pablo Díez-Villanueva, Luis Jesús Jiménez-Borreguero, Miren Uriarte-Ecenarro, Esther F. Vicente-Rabaneda, Miguel A. González-Gay, Fernando Alfonso, Santos Castañeda

**Affiliations:** 1 Cardiology Division, Hospital Universitario Príncipe de Asturias, 28805 Madrid, Spain; 2 Internal Medicine Division, Hospital Universitario Infanta Leonor, 28031 Madrid, Spain; 3 Cardiology Division, Hospital Universitario de La Princesa, IIS-Princesa, 28006 Madrid, Spain; 4 Rheumatology Division, Hospital Universitario de La Princesa, IIS-Princesa, 28006 Madrid, Spain; 5 Rheumatology Division, Hospital Universitario Fundación Jiménez Díaz, 28040 Madrid, Spain; 6 Department of Medicine, Universidad de Cantabria, 39011 Santander, Spain

**Keywords:** ankylosing spondylitis, arrhythmias, electric heart disorders, atrial fibrillation, atrioventricular block, inflammation

## Abstract

**Background/Objectives**: Ankylosing spondylitis (AS) has been associated with various comorbidities, including cardiovascular morbidity. Recent studies suggest that certain arrhythmias may be more frequent in AS patients than in the general population. The aim of this study was to analyze the prevalence of electric heart disorders (EHD) in patients with AS in real-world conditions and compare them with those reported in the general population. **Methods**: Descriptive cross-sectional study aiming to determine the prevalence of EHD in AS in pre-COVID-19 period. EHD were analyzed in a resting ECG and 24 h Holter monitoring. Additionally, the association between clinical and demographic variables was analyzed. **Results**: Among 121 patients with AS (62% men; mean ± SD age 54.6 ± 15.6 years; median [IQR] disease duration 14 (8–20) years), 18.2% presented any EHD, including 9.1% with supraventricular tachyarrhythmias (SVT) (5% atrial fibrillation [AF]) and 7.4% with atrioventricular block (AVB). Clinically relevant disorders (≥2nd-degree AVB or SVT) were observed in 9.9% of patients. In adjusted analyses, SVT was independently associated with older age and higher BMI, while any conduction delay and clinically relevant EHD were associated with age, hypertension, and disease-modifying antirheumatic-drug treatment duration. Comparisons with previous population-based studies showed similar data, with a non-significant trend toward higher AF prevalence in AS patients. **Conclusions**: There appears to be a trend toward a higher prevalence of arrhythmias in patients with AS in real-world conditions, which could have clinical and therapeutic implications. An association between EHD and pro-inflammatory conditions such as age and BMI was observed, supporting the hypothesis that underlying inflammation might contribute to increased arrhythmogenicity.

## 1. Introduction

Ankylosing spondylitis (AS) is a chronic inflammatory rheumatic disease that primarily affects the axial skeleton and frequently involves the peripheral joints, being the most common condition within the group of spondyloarthritides (SpA). The main musculoskeletal symptoms include spondylitis, oligoarthritis, and enthesitis, but extra-articular manifestations are also common [1], including uveitis (23%), inflammatory bowel disease (6.4%) [2], and cardiovascular (CV) involvement (2–10%) [3].

The prevalence of AS varies widely across studies from different countries. In Spain, it is estimated at 0.26% [4]. B27 allele of the human leukocyte antigen (HLA-B27) is present in more than 80%. In Europe, the prevalence of HLA-B27 positivity in the general population is estimated at 6–9%, while the prevalence of AS ranges from 0.08% to 0.5% [2,3,5]. The male-to-female ratio is approximately 3:1, and disease onset usually occurs before the fourth decade of life [6,7]. The pathogenesis of AS is complex, with different pathogenic mechanisms involved, along with environmental factors and biomechanical triggers [8,9,10].

The relationship between chronic inflammatory states and CV disease is well established [11,12,13] including atherothrombotic and even arrhythmic risk [13], especially atrial fibrillation (AF) [14,15]. In rheumatoid arthritis (RA), TNF-α, interleukin (IL)-6, and other proinflammatory cytokines may play a key role in arrhythmogenesis through several mechanisms [16]. These mechanisms involve remodeling of the cardiac muscle and conduction fibers, alteration of gap junctions between cardiomyocytes, and even cardiomyocyte apoptosis, which subsequently induces fibroblast activation, interfering with normal electrical impulse propagation, and generating ectopic electrical activity. Furthermore, elevated levels of C-reactive protein (CRP), IL-6, and TNF-α have been associated with a higher risk of AF [17,18]. In contrast, cardiac manifestations in AS remain a relatively unknown and poorly understood field. Mainly, aortic valve abnormalities (predominantly aortic insufficiency) and arrhythmias have been described, occurring more frequently in long-standing AS or in patients with peripheral joint involvement [3]. A European study demonstrated that patients with AS have an increased relative risk (RR) of second- and third-degree atrioventricular block (AVB) with an RR of 2.3 (95% CI 1.6–3.3), particularly in men, and of AF with an RR of 1.3 (95% CI 1.2–1.6) compared to the general population [19]. A Korean cohort reported similar associations for AF [20], findings further supported by a recent meta-analysis [21].

Finally, the effect of treating the underlying disease on the rate of CV atherothrombotic events in rheumatic diseases has been well studied, although the impact of therapy on arrhythmogenesis in these patients remains unsettled. In this context, both methotrexate (MTX) and biological therapies targeting TNF-α (TNF inhibitors) have been suggested to reduce atherothrombosis risk in RA [13]. It has also been observed that the use of anti-TNF-α therapies could stabilize or slow the progression of subclinical atherosclerosis in patients with AS [22]. Therefore, effective treatment of AS may reduce the rate of CV events (including arrhythmias) in these patients, who are generally younger than those with RA.

Currently, few studies have specifically addressed the relationship between ankylosing spondylitis and cardiac arrhythmias, and available evidence is largely derived from registry-based or administrative data with limited clinical data. Therefore, we conducted a study to evaluate the prevalence of arrhythmias and conduction disturbances in patients with AS under real-world conditions, providing clinically characterized data that may complement previous registry-based analyses.

## 2. Materials and Methods

### 2.1. Study Design

This is a cross-sectional descriptive observational study followed in the outpatient clinics of a tertiary University Hospital in Madrid, Spain. This study was conducted in accordance with current legislation and ethical standards for clinical research and was approved by the Ethics Committee of La Princesa University Hospital on 21 February 2019 (Registration number: 3677/2019). Patients recruited after 21 February 2019 were included prospectively, whereas those recruited before that date were included retrospectively. Given that arrhythmias increased overall in predisposed subjects during the SARS-CoV-2 (COVID-19) pandemic, we retrieved this study designed and carried out before 2020 to be closer to normal conditions.

### 2.2. Study Population and Protocol

**Figure 1:**
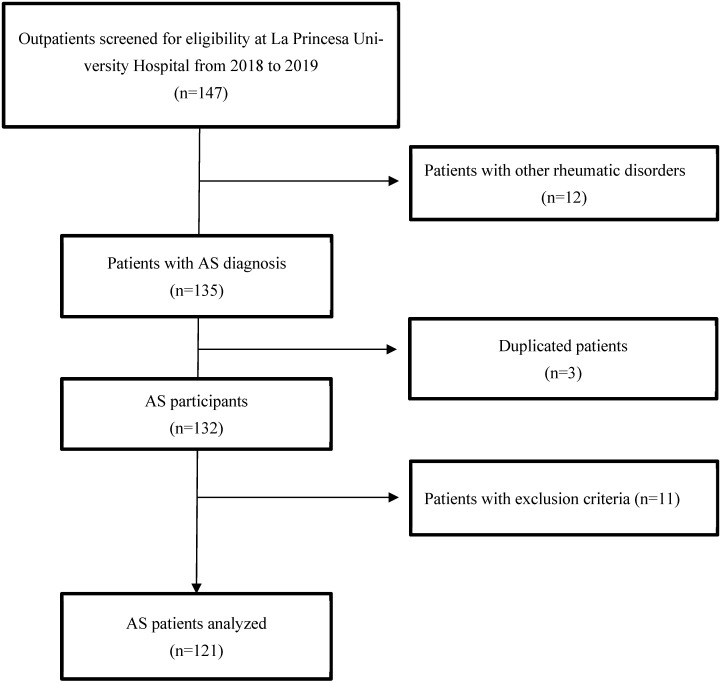
Flowchart of patients included in this study. Abbreviations: AS: Ankylosing spondylitis.

Consecutive patients diagnosed with AS according to the modified New York criteria [23] who attended the rheumatology outpatient clinics during 2018 and the first quarter of 2019, aged 18 years or older, were prospectively included in the study. A baseline 12-lead electrocardiogram (ECG, TraceMaster, Philips, Eindhoven, NL) or 24 h Holter was performed on all patients. The exact number of ECGs versus Holter recordings was not available, although Holter monitoring represented a minority of assessments. This heterogeneity of rhythm assessment may have limited the detection of intermittent arrhythmias. Patients without an ECG or other cardiac rhythm assessment (e.g., 24 h Holter) were excluded. Patient baseline characteristics were recorded by review of electronic medical records (Figure 1).

### 2.3. Electrocardiogram Analysis

A resting 12-lead surface ECG (TraceMaster, Philips, Eindhoven, NL, filter range, 0.05–150 Hz; 25 mm/s; 10 mm/mV) was performed for those who did not have one recorded after 1 January 2018.

The presence of EHD was the primary variable, and comprised supraventricular tachyarrhythmias (SVT), subcategorized as atrial fibrillation (AF) and atrial flutter or atrial tachycardia, as well as atrioventricular block, including first-degree, second-degree (Mobitz I and Mobitz II), and third-degree block, and intraventricular conduction disturbances. (hemiblocks and bundle branch blocks). Clinically relevant EHD were defined as the presence of SVT or second-degree Mobitz II or higher AVB. EHD were assessed by two blinded cardiology specialists (AGL and PDV) who evaluated all cases. In case of disagreement, a third specialist (LJJB) was checked. Diagnostic criteria followed the European Society of Cardiology guidelines from 2024 and 2021 [24,25]. Potential drug interactions as a contributing cause for AVB and intraventricular conduction disturbances were excluded. Patient medical records were also systematically reviewed for previously diagnosed EHD or treatments such as ablation or pacemaker implantation.

### 2.4. Clinical Variables

In addition, demographic, anthropometric and clinical variables were prospectively collected: sex, age, body mass index (BMI), HLA-B27 status, year of diagnosis, pattern of joint involvement, AS-related comorbidities, erythrocyte sedimentation rate (ESR) and C-reactive protein (CRP) as markers of inflammatory activity, Bath Ankylosing Spondylitis Functional Index (BASFI) and Bath Ankylosing Spondylitis Disease Activity Index (BASDAI) scores (both ranging from 0 to 100), regular daily physical activity, smoking status, and current treatment. Regular physical activity was defined as performing at least 150 min of moderate-intensity exercise per week, assessed either through self-reported questionnaires or clinical interviews. Additionally, the presence of other arrhythmogenic comorbidities was recorded, including thyroid dysfunction, chronic obstructive pulmonary disease, obstructive sleep apnea–hypopnea syndrome, type 2 diabetes mellitus, hypertension, and ischemic heart disease.

### 2.5. Statistical Analysis

All continuous variables were tested for normality using the Kolmogorov–Smirnov and Shapiro–Wilk tests. Variables following a normal distribution are reported as mean ± standard deviation (SD) and were compared between two groups using the unpaired Student’s *t* test, or by one-way analysis of variance (ANOVA) when >2 groups were analyzed. Non-normally distributed variables are presented as median and interquartile range (IQR) and compared using the Mann–Whitney U test (two groups) or the Kruskal–Wallis test (multiple groups). Discrete and categorical variables are expressed as absolute frequencies and percentages. Comparisons between groups were performed using the χ^2^ test. When expected frequencies in contingency tables were <5, Fisher’s exact test was applied.

Univariate logistic regression analyses were used to determine odds ratios (OR) and 95% CI for associations between all recorded variables and outcomes. Variables with a significance threshold of *p* < 0.20 in univariate analyses were entered as covariates into a multivariate logistic regression model. A stepwise backward selection procedure with a *p* value of 0.05 was used to retain the most strongly associated variables. For comparisons with prior prevalence registries, absolute differences in prevalence proportions were estimated and tested using standard two-sample proportion methods. All statistical tests were two-tailed, with *p* < 0.05 considered as statistically significant. Statistical analyses were performed using IBM SPSS Statistics version 27.0 (IBM Corp., Armonk, NY, USA) and Stata version 17 (StataCorp LLC, College Station, TX, USA).

## 3. Results

### 3.1. Baseline Clinical Features of the Patients

Initially, 147 patients were preselected, of whom 135 had been diagnosed with AS. Among these 135 patients, three were duplicates in the database, and a total of 11 subjects did not have a baseline ECG and were therefore excluded from the study. Thus, the final sample size was 121 patients (Figure 1). Of them, seventy-six patients were men (62.8%) and the mean age was 54.6 years. Median disease duration was 14 [IQR: 8–20] years. Regarding inflammatory activity parameters, the median ESR and CRP values were 8 [IQR: 4–21] mm/h, and 0.3 [IQR: 0.1–0.6] mg/dL, respectively. The median BASFI and BASDAI scores were 26/100 [IQR:7–39] and 28/100 [IQR: 6–55], respectively.

More than 82% of patients were receiving some type of disease-modifying antirheumatic drug (DMARD), with biological agents being the most used (50.4%), while 49.6% were regular users of nonsteroidal anti-inflammatory drugs (NSAIDs). Only six patients (5.1%) were not on any specific pharmacological treatment. Median treatment duration was 4.5 [IQR: 0–11.5] years. The mean (±SD) BMI was 26.9 (±4.8) kg/m^2^, and 76.5% of patients engaged in regular daily physical activity (at least 150 min of moderate-intensity exercise per week, as previously defined). The proportion of smokers was 20.7% (Table 1).

### 3.2. Electric Heart Disorders

Of the 121 patients studied, 22 presented any EHD, resulting in an estimated overall AS prevalence of EHD of 18.2 (95% CI 11.3–25.1%).

Analyzing each type of EHD separately, 11 patients (9.1%) presented SVT with AF specifically accounting for 5%. Additionally, one patient had symptomatic intranodal reentrant tachycardia (IRT) (0.9%), which required an ablation procedure, but this was not considered for the analysis, as pathophysiology differs substantially. AVB of any degree was found in nine patients (7.4%), most of them being suprahissian. Intraventricular conduction delays were detected in seven (5.8%) cases, most of them with wide QRS complex. Finally, clinically relevant EHD (including infrahissian AV blocks and SVT) were found in 12 patients (9.9%) (Table 2).

In the unadjusted analysis, older age, hypertension and higher BMI were associated with SVT, whereas in the adjusted model, only age and BMI remained significantly associated with SVT, mostly driven by AF (Table 3).

For any conduction delay, univariate analysis also identified older age, hypertension, and longer treatment duration as significant risk factors, while in the adjusted analysis, only hypertension and treatment duration remained independently associated (Table 4).

**Table 4:** Logistic regression model for any conduction delay (n = 16) in AS patients (n = 121).

Variables	Unadjusted Analysis			Adjusted Analysis		
	OR	95% CI	*p*-Value	OR	95% CI	*p*-Value
Age (years)	1.06	1.01–1.10	0.008			
Male sex	2.37	0.62–9.00	0.205			
Type 2 diabetes mellitus	3.40	0.59–19.47	0.169			
Hypertension	6.13	1.79–21.02	0.004	6.1	1.71–21.76	0.005
Disease duration (years)	1.05	0.99–1.11	0.111			
ESR, mm/1st h	1.02	0.99–1.05	0.217			
CRP, mg/dL	0.87	0.43.1.72	0.682			
DMARDs	0.74	0.19–2.93	0.670			
Treatment duration (years)	1.06	1.00–1.13	0.037	1.07	1.00–1.13	0.043
BMI, kg/m^2^	1.07	0.95–1.19	0.292			

BMI: body mass index; CRP: C-reactive protein; DMARDs: disease-modifying antirheumatic drugs; ESR: erythrocyte sedimentation rate. Regarding any relevant EHD (≥2nd-degree Mobitz II AVB or SVT), univariate analysis showed significant associations with age, hypertension, and BMI. In the adjusted analysis, only age remained significant, whereas BMI showed a borderline association (OR 1.15, 95% CI 0.99–1.33, *p* = 0.071) (Table 5). Because of the relatively small number of events, multivariable models may be prone to modest overfitting, which should be considered when interpreting the results.

**Table 5:** Logistic regression model for any relevant electric heart disorder (>2nd degree Mobitz II or supraventricular tachyarrythmia [n = 12]) in AS patients (n = 121).

Variables	Unadjusted Analysis			Adjusted Analysis		
	OR	95% CI	*p*-Value	OR	95% CI	*p*-Value
Age (years)	1.07	1.02–1.12	0.005	1.07	1.01–1.14	0.013
Male sex	0.38	0.11–1.29	0.120			
Type 2 diabetes mellitus	4.16	0.71–24.27	0.113			
Hypertension	4.61	1.30–16.37	0.018			NS
Disease duration (years)	1.00	0.93–1.07	0.979			
ESR, mm/1st h	0.97	0.90–1.04	0.341			
CRP, mg/dL	1.00	0.97–1.04	0.804			
DMARDs	1.16	0.74–1.81	0.529			
Treatment duration (years)	0.37	0.10–1.37	0.136			
BMI, kg/m^2^	1.17	1.02–1.34	0.023	1.15	0.99–1.33	0.071

BMI: body mass index; CRP: C-reactive protein; DMARDs: disease-modifying antirheumatic drugs; ESR: erythrocyte sedimentation rate; NS: non-significant.

### 3.3. Comparison with Previous Studies

The prevalence of arrhythmias in our cohort was compared with previous studies (Table 6) [19,26,27]. Compared to the OFRECE study also conducted in Spain [26], which includes subjects from the general population, the difference in AF prevalence was 0.6% (95% CI −3.3–4.5), which was not statistically significant (*p* = 0.78). A comparison with a Swedish cohort of AS patients [19] and the general population showed a difference in AF prevalence of 2.2% (95% CI −2.0–6.3), also not significant. Regarding second- to third-degree AVB, the difference compared to the general population was 0.5% (95% CI −1.1–2.1; *p* = 0.52). Finally, the prevalence of first-degree AVB compared to a Finnish study in healthy subjects showed a difference of 2.0% (95% CI −1.5–5.6; *p* = 0.26), which was not significant either.

## 4. Discussion

Early diagnosis and management of extra-articular manifestations, alongside musculoskeletal symptoms control, remains a cornerstone in AS patients’ care. While some clinical manifestations are well characterized, others are less understood. This study provides insight into the epidemiology of heart rhythm disorders in AS, revealing a notable prevalence of EHD, with AVB and AF being the most frequently observed.

A key finding is the significant association between BMI and EHD. Overweight and obesity increased adiposity may enhance a proinflammatory microenvironment through increased production of adipokines such as leptin, visfatin, and resistin, which have been linked to elevated CRP, TNF-α, and IL-6 levels [28,29,30]. In our cohort, however, elevated CRP levels or other inflammation biomarkers were not associated with EHD, which might be related to a relatively stable outpatient population and the limited sample size. More sensitive inflammatory assessments, such as high-sensitivity CRP or other specific biomarkers, may be able to detect subtle inflammation. Obesity has also been implicated as a risk factor for autoimmune rheumatic diseases, including RA, AS, and psoriasis-associated arthritis [30,31,32]. This, combined with the baseline inflammatory state inherent to AS, supports the hypothesis that inflammation may play a direct role in arrhythmogenesis in these patients [29]. Indeed, CRP and IL-6 have been proposed as biomarkers for AF risk stratification [33]. Several potential mechanisms have been described, including sodium channel hyperactivation, altered calcium handling in cardiomyocytes, myocardial fibrosis, increased cardiac automatism, and the formation of reentry circuits. Notably, glucocorticoids and NSAIDs have been shown to reduce the incidence of post-coronary artery bypass grafting AF, highlighting possible therapeutic implications [34].

Considering all these aspects, the association observed between age and the presence of AF can also be explained. Aging has been linked to dysregulation of cytokine networks and the establishment of a proinflammatory environment mediated, among other pathways, by IL-1β and NF-κB mediators. This phenomenon has been termed by some authors as “inflammaging” [35].

Interestingly, treatment duration was also observed in patients with any conduction delay, an association that should be interpreted with caution as it is likely influenced by age, depends on the time of diagnosis and might be longer in those with more severe stages of the disease, rather than reflecting a direct effect of treatment.

Other morbidities collected in our study such as obstructive sleep apnea–hypopnea syndrome, hypertension, ischemic heart disease, type 2 diabetes mellitus, or chronic obstructive pulmonary disease, are also recognized risk factors for arrhythmias [36,37,38] but were not associated with arrhythmia occurrence in our cohort, probably due to the small sample size.

One of the most positive aspects of this work is that it was performed in “real-world conditions”, understood as unselected inclusion of consecutive outpatients attending routine clinical care before the COVID-19 pandemic. Indeed, during the recent pandemic, an increase in arrhythmias was reported, likely related to systemic inflammation especially in predisposed subjects [39,40]. Therefore, we retrieved this study designed and carried out before the SARS-CoV-2 (COVID-19) pandemic, including more realistic and usual epidemiological conditions.

Finally, we decided to compare our series with others previously described in the literature. The results obtained regarding prevalence were similar with previous reports. However, our cohort may differ in demographic characteristics including age, sex and region, diagnostic methods and comorbidities. The OFRECE study, conducted in Spain to estimate the prevalence of AF, included subjects from the general population aged over 40 years [26]. The difference in AF prevalence between our series and this study was 0.6%, which was not statistically significant. Additionally, our results were compared to those of a study conducted in Sweden [19] which determined the prevalence of arrhythmias in subjects with AS and in the general population aged over 18 years. The difference in AF prevalence between our study and the Swedish cohort was 2.2%, not statistically significant either. In contrast, the Swedish study found a significant 1.3% higher AF prevalence in patients with AS compared to the general population. Regarding second- to third-degree AV block, the difference in prevalence between our AS patients and the general population was 0.5%, again not significant. On the contrary, the Swedish series reported a similar 0.6% difference, which resulted statistically significant. Finally, the prevalence of first-degree AVB was compared to a study conducted in Finland with healthy subjects aged 30–59 years [27]. The difference in first-degree AVB prevalence between our series and this study was 2.0%, which did not show significant differences. Although first-degree AVB is traditionally considered benign, accumulating evidence, including a recent meta-analysis, indicates that it is associated with an increased risk of all-cause mortality, AF, and heart failure [41]. Progression from first-degree AVB to higher-degree block may be influenced by underlying conditions, including chronic inflammation [41].

The main limitations of this study are the small absolute sample size, its cross-sectional and unicentric design, and the lack of a matched control group from the same population. However, the relatively wide confidence intervals observed in several multivariable estimates reflect limited precision, likely related to the modest sample size and low number of events. The use of a single baseline ECG in most of the subjects may certainly underestimate arrhythmia prevalence, representing a potential source of underdetection of intermittent arrhythmias. A minority of patients without ECG or Holter monitoring were excluded. Although the absence of ECG or Holter assessment was mainly due to organizational or logistical reasons rather than clinical criteria or disease severity, detailed clinical profiles of these patients were not available, which may have introduced selection bias. Additionally, the number of patients who underwent standard ECG or 24 h Holter monitoring, as well as the proportion of arrhythmia diagnoses specifically derived from Holter recordings, was not available. Conversely, this highlights the value of our findings, as more sensitive diagnostic methods—such as 24 or 72 h Holter monitoring or electrophysiological studies—would likely detect a higher prevalence of arrhythmia and EHD. In contrast, patients included in this study were followed in a hospital setting, maybe reflecting more severe AS phenotypes and potentially overestimating prevalence rates. Additional limitations include the unavailability of echocardiographic data and of more specific immunological assessments for patients with EHD, as well as the lack of control group. Comparisons with previous registry-based studies may be limited by differences in demographic characteristics (including age, sex, and region), diagnostic methods, and comorbidities, which may affect the internal validity of these comparisons and represent a notable limitation of the study.

## 5. Conclusions

Our findings, together with previous reports, suggest a trend toward higher arrhythmia prevalence in AS compared to the general population. In our study, designed in real-world conditions in pre-COVID-19 period, arrhythmias were associated with hypertension, older age, and higher BMI. The latter two factors are linked to systemic inflammation, which, together with the inflammatory microenvironment in AS, may promote EHD. These results support the hypothesis that inflammation contributes to arrhythmogenesis in AS. Routine and periodic ECG screening, careful evaluation of arrhythmias during concomitant events, and collaboration between rheumatology and cardiology teams may be considered, particularly in at-risk patients, appear to be useful strategies to help optimize early detection and management of arrhythmias in individuals with AS.

## Figures and Tables

**Table 1:** Baseline clinical characteristics of the study group of patients with AS (n = 121).

Variable	Overall Cohort (n = 121)	Arrythmia (n = 11)	No Arrythmia (n = 110)	*p*-Value	Conduction Delays (n = 14)	No Conduction Delays (n = 107)	*p*-Value
Male sex, n (%)	76 (62.8)	4 (36.4)	72 (65.5)	0.098	11 (78.6)	65 (60.8)	0.248
Age (years)	54.6 ± 15.6	66.5 ± 16.4	53.4 ± 15.1	0.003	65.4 ± 13.6	53.1 ± 15.4	0.003
BMI, kg/m^2^	26.9 ± 4.8	30.3 ± 6.0	26.5 ± 4.5	0.009	28.2 ± 4.2	26.7 ± 4.9	0.146
Current smokers, n (%)	25 (20.7)	0 (0.0)	25 (22.7)	0.075	1 (7.1)	24 (22.4)	0.223
Daily physical activity, n (%)	88 (76.5)	9 (81.8)	79 (76.0)	1	9 (10.2)	79 (88.7)	0.5
HLA B27 positive	92 (82.1)	9 (81.8)	83 (82.2)	1	12 (85.7)	80 (81.6)	1
AS-related comorbidities							
-Uveitis, n (%)	19 (15.7)	1 (9.1)	18 (16.4)	0.485	2 (14.3)	17 (15.9)	1
-Inflammatory bowel disease, n (%)	13 (10.7)	0 (0.0)	13 (11.8)	0.601	1 (7.1)	12 (11.2)	1
Disease duration (years)	14 (8–20)	9 (6–17)	14 (8–21)	0.194	20.5 (19–23)	14 (7–20)	0.024
Disease activity parameters							
-ESR, mm/1st h, median (IQR)	8 (4–21)	6 (5–15)	8 (4–21.5)	0.883	10.5 (4–24)	8 (4–21)	0.801
-CRP, mg/dL, median (IQR)	0.3 (0.1–0.6)	0.2 (0.1–0.5)	0.3 (0.1–0.6)	0.686	0.2 (0.1–0.5)	0.3 (0.1–0.6)	0.394
-BASFI (0–100)	26 (7–39)	3.5 (0–4.5)	2.6 (0.7–3.7)	0.782	3.5 (1.9–4.5)	2.4 (0.6–3.6)	0.133
-BASDAI (0–100)	28 (6–55)	4 (0–6.1)	2.8 (0.6–4.9)	0.785	3.4 (1.4–5.7)	2.7 (0.6–5.1)	0.636
Current treatment							
DMARDs, n (%)	100 (82.6)	7 (63.64)	93 (84.6)	0.098	11 (78.6)	89 (83.2)	0.709
Sulfasalazine, n (%)	58 (47.9)	3 (27.3)	55 (50)	0.209	4 (28.6)	54 (50.5)	0.159
Methotrexate, n (%)	23 (19.0)	3 (27.3)	20 (18.2)	0.436	3 (21.4)	20 (18.7)	0.729
Biological drugs, n (%)	61 (50.4)	4 (36.4)	57 (51.8)	0.363	8 (57.1)	53 (49.5)	0.592
Glucocorticoids, n (%)	5 (4.1)	0 (0.0)	5 (4.6)	1	1 (7.1)	4 (3.7)	0.465
NSAIDs, n (%)	60 (49.6)	8 (72.7)	52 (47.3)	0.126	5 (35.71)	55 (51.4)	0.270
Treatment duration (years)	4.5 (0–11.5)	4 (1–9)	5 (0–12)	0.913	12 (1–20)	4 (0–11)	0.040
Other comorbidities							
Ischemic heart disease, n (%)	7 (5.8)	1 (9.1)	6 (5.5)	0.622	1 (7.1)	6 (5.6)	0.587
Hypertension, n (%)	41 (33.9)	7 (63.6)	34 (30.9)	0.043	10 (71.4)	31 (29.0)	0.002
COPD, n (%)	3 (2.5)	0 (0.0)	3 (2.7)	1	1 (7.1)	2 (1.9)	0.311
OSAHS, n (%)	9 (7.5)	1 (9.1)	8 (7.3)	0.589	1 (7.1)	8 (7.5)	1
Type 2 diabetes, n (%)	7 (5.8)	1 (9.1)	6 (5.5)	0.496	2 (14.3)	5 (4.7)	0.186

Data are presented as n (%) of the total, mean ± standard deviation or median (interquartile range) as appropriate. AS: ankylosing spondylitis; BASDAI: Bath Ankylosing Spondylitis Disease Activity Index; BASFI: Bath Ankylosing Spondylitis Functional Index; BMI: body mass index; COPD: chronic obstructive pulmonary disease; CRP: C-reactive protein; DMARD: disease-modifying antirheumatic drug; ESR: erythrocyte sedimentation rate; HLA: human leukocyte antigen; NSAID: non-steroidal anti-inflammatory drug; OSAHS: obstructive sleep apnea–hypopnea syndrome.

**Table 2:** Electric heart disorders in AS patients (n = 121).

Electric Heart Disorders in AS Patients	N (%)
Supraventricular tachyarrhythmias	
Atrial fibrillation	6 (5)
Atrial flutter or atrial tachycardia	5 (4.1)
Atrioventricular conduction delay	
First degree AVB or second degree Mobitz I AVB	8 (6.6)
Second degree Mobitz II or third degree AVB	1 (0.8)
Intraventricular conduction delay	
With QRS < 120 ms	2 (1.7)
With QRS > 120 ms	5 (4.1)

AS: ankylosing spondylitis; AVB: atrioventricular block.

**Table 3:** Logistic regression model for supraventricular tachyarrhythmias (n = 11) in AS patients (n = 121).

Variables	Unadjusted Analysis			Adjusted Analysis		
	OR	95% CI	*p*-Value	OR	95% CI	*p*-Value
Age (years)	1.06	1.01–1.11	0.011	1.07	1.01–1.13	0.017
Male sex	0.30	0.8–1.1	0.069			
Type 2 diabetes mellitus	1.73	0.19–15.9	0.626			
Hypertension	3.91	1.07–14.3	0.039			
Disease duration (years)	0.95	0.88–1.02	0.184	0.92	0.84–1.01	0.080
ESR, mm/1st h	1.00	0.96–1.04	0.919			
CRP, mg/dL	1.19	0.76–1.86	0.442			
DMARDs	0.32	0.08–1.21	0.094			
Treatment duration (years)	0.98	0.90–1.06	0.573			
BMI, kg/m^2^	1.17	1.02–1.34	0.028	1.18	1.01–1.40	0.038

BMI: body mass index; CRP: C-reactive protein; DMARDs: disease-modifying antirheumatic drugs; ESR: erythrocyte sedimentation rate.

**Table 6:** Comparison of the prevalence of heart rhythm disorders in our patients with AS compared to those previously published in the literature.

Electric Heart Disorders	n	(%)	Prevalence Differences ^3^	95% CI of Difference	*p*-Value
**AF in general population ^1^** [26]	8343	4.4	Reference		
-AF in AS (present series)	121	5.0	0.6%	−3.3–4.5	0.78
**AF in general population** [19]	266,435	2.8	Reference		
-AF in AS [19]	6448	4.1	1.3%	0.8–1.8	<0.001
-AF in AS (present series)	121	5.0	2.2%	−2.0–6.3	0.31
**AVB grade II–III in general population** [19]	266,435	0.3	Reference		
-AVB grade II–III in AS [19]	6448	0.9	0.6%	0.8–0.4	<0.001
-AVB grade II–III in AS (present series)	121	0.8	0.5%	−1.1–2.1	0.52
**AVB grade I in general population ^2^** [27]	10,785	2.1	Reference		
-AVB grade I in AS (present series)	121	4.1	2.0%	−1.52–5.58	0.26

Notes: n, sample size; %, prevalence. Comparisons are made with historical cohorts from previous registry-based studies. ^1^ The OFRECE study included subjects aged ≥ 40 years. ^2^ The Aro et al. study included subjects aged 30–59 years. ^3^ For Bengtsson et al. cohort study, end-of-follow-up prevalence was used for comparison, as these studies reported incidence. AF: atrial fibrillation; AS: ankylosing spondylitis; AVB; atrioventricular block; CI: confidence interval.

## Data Availability

Due to the sensitive nature of clinical data, the datasets generated and analyzed during the current study are not publicly available. However, they can be obtained from the corresponding authors upon reasonable request.

## Abbreviations

AF	Atrial fibrillation
AS	Ankylosing spondylitis
AVB	Atrioventricular block
BASDAI	Bath Ankylosing Spondylitis Disease Activity Index
BASFI	Bath Ankylosing Spondylitis Functional Index
BMI	Body mass index
CI	Confidence interval
CRP	C-reactive protein
DMARDs	Disease-modifying antirheumatic drugs
ECG	Electrocardiogram
EHD	Electric heart disorders
ESR	Erythrocyte sedimentation rate
HLA	Human leukocyte antigen
IL	Interleukin
IQR	Interquartile range
IRT	Intranodal reentrant tachycardia
NSAID	Nonsteroidal anti-inflammatory drug
RR	Relative risk
SpA	Spondyloarthritis/spondyloarthritides
SVT	Supraventricular tachyarrhythmia
TNF-α	Tumor necrosis factor alpha
